# A prospective cohort examination of haematological parameters in relation to cancer death and incidence: the Busselton Health Study

**DOI:** 10.1186/s12885-018-4775-x

**Published:** 2018-09-03

**Authors:** Niwansa Adris, Anita Chai Geik Chua, Matthew William Knuiman, Mark Laurence Divitini, Debbie Trinder, John Kevin Olynyk

**Affiliations:** 1 Department of Gastroenterology and Hepatology, Fiona Stanley and Fremantle Hospital Group, Murdoch, WA 6150 Australia; 2 Medical School, The University of Western Australia, Fiona Stanley Hospital, Murdoch, WA 6150 Australia; 3 Harry Perkins Institute of Medical Research, Murdoch, WA 6150 Australia; 4 School of Population and Global Health, The University of Western Australia, Crawley, WA 6009 Australia; 5 School of Health and Medical Sciences, Edith Cowan University, Joondalup, 6027 Western Australia

**Keywords:** Iron, Full blood count, Cancer

## Abstract

**Background:**

Cancer risk is associated with serum iron levels. The aim of this study was to evaluate whether haematological parameters reflect serum iron levels and may also be associated with cancer risk.

**Methods:**

We studied 1564 men and 1769 women who were enrolled in the Busselton Health Study, Western Australia. Haematological parameters evaluated included haemoglobin (Hb), mean cell volume (MCV), mean cell haemoglobin (MCH) and mean cell haemoglobin concentration (MCHC) and red cell distribution width (RCDW). Statistical analyses included t-tests for quantitative variables, chi-square tests for categorical variables and Cox proportional hazards regression modelling for cancer incidence and death.

**Results:**

There was marginal evidence of an association between MCV (as a continuous variable) and non-skin cancer incidence in women (HR 1.15, 95% CI 1.013, 1.302; *p* = 0.030) but the hazard ratio was attenuated to non-significance after adjustment for serum ferritin (SF), iron and transferrin saturation (TS) (HR 1.11, 95% CI 0.972, 1.264; *p* = 0.126). There was strong evidence of an association between MCHC and prostate cancer incidence in men; the estimated hazard ratio for an increase of one SD (0.5) in MCHC was 1.27 (95% CI 1.064, 1.507; *p* = 0.008). These results remained significant after further adjustment for SF and iron; the estimated hazard ratio for an increase of one SD (0.5) in MCHC was 1.25 (*p* = 0.014, 95% CI 1.05 to 1.48).

**Conclusions:**

The MCHC and MCV were associated with cancer incidence in a Western Australian population, although only MCHC remained associated with prostate cancer after adjusting with serum iron and TS (circulating iron) and SF (storage iron). Haematological parameters are thus of limited utility in population profiling for future cancer risk.

## Background

Iron is an essential micronutrient for human health as it participates in a vast range of metabolic processes [1–4]. Deficiency or excess of iron have both been implicated as vital pathogenic processes of various chronic diseases. Iron deficiency is implicated in anaemia [5, 6], worsening symptoms of chronic heart failure [7, 8] and restless leg syndrome [9, 10]. On the other hand, iron excess as observed in hereditary haemochromatosis and haematological disorders such as thalassaemia major and sickle cell disease has been associated with liver cirrhosis, type two diabetes mellitus and cardiomyopathy [11–20].

Recent studies have implicated iron in the pathogenesis of cancer. In hereditary haemochromatosis an increased risk of cancer with iron overload has been demonstrated [21–24]. More recent population studies have shown even at high physiological levels of iron, there was increased risk of cancer [25–27]. On the contrary, depletion of iron has been proposed to have a protective role on cancer development [28–35]. In our previous study of the Busselton population, we found that higher concentrations of serum iron or TS were associated with an increased incidence of non-skin cancer in women, increased risks of breast cancer and of cancer death [25]. Thus iron status may be useful in stratifying risk for cancer.

Iron is essential for erythropoiesis [36], and low serum iron parameters may be reflected by changes in haematological parameters such as a reduction in mean haemoglobin (Hb), mean corpuscular volume (MCV), mean cell haemoglobin (MCH), and mean corpuscular haemoglobin concentration (MCHC) and an increase in red cell distribution width (RCDW) [37, 38]. Conversely, several studies of individuals with iron overload, such as in hereditary haemochromatosis, have shown that elevation in iron stores was associated with increased values of Hb, MCV, MCH, and MCHC [39–41].

The full blood count assay is a relatively cheap and easily measured laboratory investigation that is conducted worldwide. In Australia it is estimated that approximately 12 million full blood counts are ordered per year [42]. Whilst serum iron biochemistry is also commonly performed, the full blood count is performed more than twice as frequently as iron studies [42]. The cost of the full blood count is approximately half the cost of iron studies in Australia and elsewhere [43–45].

Given the positive association between iron levels and cancer incidence and death from our previous study [25], we aimed to assess the utility of haematological parameters such as the Hb, RCDW, MCV, MCH and MCHC as surrogate markers of iron bioavailability to determine the associations between these parameters and cancer death and incidence in the Busselton population.

## Methods

### Study population

Busselton is a city situated in the southwest of Western Australia and more than 90% of its residents consist of individuals with Anglo-Celtic ancestry. The residents of this coastal city have been regularly surveyed since the introduction of the Busselton Health Study in 1966. A follow-up health survey of survivors from surveys during 1966 to 1987 was performed in 1994 and 1995 [46, 47]. Inclusion criteria for this study were individuals who participated in the 1994/1995 survey aged 25–79 years and who had relevant data, no history of cancer at the time of survey, not taking haemopoietic agents and SF ≥20 μg/L. This study was approved by the Busselton Population Medical Research Institute and ethics approval was obtained from the Human Research Ethics Committee of the Health Department of Western Australia (Project number 2011/60).

### Clinical and biochemical measurements

Participants of this survey completed a comprehensive health and lifestyle questionnaire and were subject to various measurements and tests [46]. Data on smoking, alcohol intake, menopausal status, use of iron supplements, and history of blood donations was obtained from the questionnaire. Alcohol use was classified as light, moderate and heavy if intake was < 140 g/week, 140–420 g/week and > 420 g/week, respectively. Trained assessors obtained data on anthropometric measures such as waist circumference, weight and height, from which body mass index (BMI) was derived. Blood pressures were measured after five minutes of rest in a seated position.

After an overnight fast at time of the survey, blood samples were collected from the participants. Their serum was separated and stored at − 70 °C. Serum biochemical measurements of haematological indices (Hb, MCV, MCH, MCHC, RCDW), iron indices (iron, TS, SF), lipids (high density lipoprotein (HDL) cholesterol, triglyceride), glucose and insulin, liver function enzymes and proteins (alanine transaminase (ALT), gamma-glutamyl transferase (GGT), albumin, bilirubin) and a marker of inflammation (C-reactive protein; C-RP) were performed using standard protocols by PathWest Laboratories (Nedlands, Western Australia). Using the following equation: (fasting insulin (μIU/mL) x fasting glucose (mmol/L)) ÷ 22.5, the Homeostasis Model Assessment-estimated insulin resistance (HOMA-IR) score was calculated.

### Cancer outcomes

By accessing the Death Register and Cancer Register from the Western Australian Department of Health within the time frame of January 1994 until June 2014, the incidence of cancer and death records were collected [48]. Cancer variables analysed included death from (non-skin) cancer (ICD10 C00-C42, C45-C97), incident non-skin cancer (ICD10 C00-C42, C45-C97), incident prostate cancer (men only, ICD10 C61), incident breast cancer (women only, ICD10 C50), and incident colorectal cancer (ICD10 C18-C21) where an incident case is a non-fatal or fatal case.

### Statistical analyses

Statistical analyses were performed using SAS® 9.4. Log transformation of data was performed for variables that had positively skewed distributions (SF, iron and TS, triglycerides, glucose, HOMA-IR, ALT, GGT, C-RP). Differences in characteristics between men and women were examined using t-tests for quantitative variables and chi-square tests for categorical variables. The associations between haematological parameters (Hb, MCV, MCH, MCHC and RCDW) and cancer incidence and death were examined using Cox proportional hazards regression modeling. Haematological parameters were examined both as a continuous variable and in three gender-specific approximate tertile categories. The estimated hazard ratios with 95% confidence interval were reported for each of the haematological parameters in relation to cancer incidence and death in men and women based on cancer and mortality follow-up to 30 June 2014. 95% confidence intervals for a hazard ratio that do not include the value 1 are significant at the 5% level (i.e. *p* <  0.05). All models were adjusted for age, smoking, alcohol consumption, BMI, and waist circumference. Modelling of breast cancer risk in women was also adjusted for menopausal status.

## Results

A total of 1564 men and 1769 women met the study inclusion criteria. Baseline demographic, anthropometric and biochemical characteristics of the cohort, stratified according to gender, are presented in Table 1. The study cohort comprised of 47% men, with a mean age of 51 ± 14 years, and 53% of women with a mean age of 52 ± 15 years. Approximately 57% of women were post-menopausal and a third of these women used hormone replacement therapy. Oral contraceptives were used by 27% of pre-menopausal women. More men were current smokers or ex-smokers and also consumed more alcohol than women. A small proportion of the Busselton cohort was taking iron supplements to treat iron deficiency, 0.6% of men and 4.7% of women respectively, and about a third of men and women reported a history of blood donation. Hb and MCHC were significantly higher in men (*p* <  0.001) whereas MCV, MCH and RCDW were similar in men and women. SF, iron and TS levels were higher in men than in women (*p* <  0.001) and had positively skewed distributions. With the exception of HDL cholesterol and C-RP levels, all the other measured variables were also higher in men than in women (*p* <  0.001).

**Table 1:** Characteristics of the study cohort and number of cancers and cancer deaths by gender. Table shows mean (SD), percent or number (percent) for cancer outcomes

Characteristic	Men (*n* = 1564)	Women (*n* = 1769)	*p*-value
Hb (g/dL)	151.4 (9.6)	135.5 (8.7)	< 0.001
MCV (fL)	89.0 (3.6)	89.1 (3.7)	0.225
MCH (pg/cell)	30.6 (1.3)	30.5 (1.4)	0.152
MCHC (g/dL)	34.4 (0.5)	34.3 (0.5)	< 0.001
RCDW	0.13 (0.01)	0.13 (0.01)	0.014
SF (μg/L)	228 (211)	101 (105)	
Log SF	5.17 (0.73)	4.33 (0.73)	< 0.001
Serum iron (μmol/L)	19.0 (5.6)	17.9 (5.7)	
Log serum iron	2.90 (0.30)	2.83 (0.33)	< 0.001
TS (%)	30.2 (10.5)	27.0 (13.9)	
Log TS	3.35 (0.34)	3.23 (0.36)	< 0.001
Age (years)	50.7 (14.4)	51.9 (15.1)	0.022
Smoking status			
Never	41.6	58.5	< 0.001
Ex	41.8	30.0	
Current	16.6	11.6	
Alcohol consumption Never	3.3	8.2	< 0.001
Ex	6.4	11.6	
Light	48.5	67.2	
Moderate/Heavy	39.1	8.6	
Unknown	2.7	4.4	
Menopausal status Pre/OC No	–	31.4	–
Pre/OC Yes	–	11.6	
Post/HRT No	–	38.4	
Post/HRT Yes	–	18.5	
Use of iron supplement	0.6	4.7	< 0.001
History of blood donation	34.6	28.9	< 0.001
Body mass index (kg/m^2^)	26.7 (3.3)	25.9 (4.7)	< 0.001
Waist circumference (cm)	93.6 (9.9)	81.2 (11.8)	< 0.001
Systolic blood pressure (mm Hg)	127 (15)	122 (18)	< 0.001
Diastolic blood pressure (mm Hg)	78 (10)	73 (10)	< 0.001
High density lipoprotein chol (mmol/L)	1.21 (0.30)	1.54 (0.39)	< 0.001
Triglycerides (mmol/L)	1.47 (1.06)	1.21 (0.75)	
Log Triglycerides	0.21 (0.56)	0.05 (0.52)	< 0.001
Glucose, mmol/L	5.13 (1.43)	4.90 (1.22)	
Log Glucose	1.61 (0.18)	1.57 (0.17)	< 0.001
HOMA-IR	1.90 (5.11)	1.67 (2.13)	
log HOMA-IR	0.33 (0.68)	0.24 (0.65)	< 0.001
ALT (IU/L)	28.2 (16.3)	19.3 (10.6)	
Log ALT	3.23 (0.45)	2.86 (0.41)	< 0.001
GGT (IU/L)	31.0 (23.8)	21.8 (17.2)	
Log GGT	3.27 (0.53)	2.93 (0.50)	< 0.001
Bilirubin (μmol/L)	11.2 (5.3)	9.0 (3.8)	< 0.001
Albumin (g/L)	45.9 (2.7)	44.7 (2.6)	< 0.001
C-RP (mg/L)	2.69 (7.30)	3.62 (10.08)	
log C-RP	0.26 (1.18)	0.54 (1.22)	< 0.001
Cancer outcomes			
Non-skin cancer death	94 (6.0)	97 (5.5)	0.514
Non-skin cancer	304 (19.4)	284 (16.1)	0.011
Prostate cancer	129 (8.2)	–	–
Breast cancer	–	92 (5.2)	–
Colorectal cancer	42 (2.7)	46 (2.6)	0.878

*OC* (oral contraceptives), *HRT* (hormone replacement therapy), *HOMA-IR* (Homeostasis Model Assessment – estimated insulin resistance), *ALT* (alanine transaminase), *GGT* (gamma-glutamyltransferase), *C-RP* (C-reactive protein)

A total of 202 cohort participants died from cancer during the 20-year follow-up period and 191 were non-skin cancer deaths. A total of 588 participants had an incident non-skin cancer. A greater proportion of men developed non-skin cancer compared to women (19.4% in men versus 16.1% in women) whilst a similar proportion of non-skin cancer deaths and incident colorectal cancers were recorded for both men and women during the follow-up period. Of the sex-specific cancers, the proportion of men who were diagnosed with prostate cancer and women who were diagnosed with breast cancer was 8.2% and 5.2%, respectively.

Table 2 shows the correlations between haematological parameters and iron parameters. Hb was weakly correlated with MCV and moderately positively correlated with MCH and MCHC (0.04 to 0.14). MCV and MCH were very strongly positively correlated (0.93 in men and women), however MCV was not correlated with MCHC whereas MCH was positively correlated with MCHC (0.30 in men and 0.36 in women). RCDW was weakly to moderately negatively correlated with Hb, MCV, MCH, and MCHC.

**Table 2:** Correlations between haematological parameters and iron parameters in men and women

	Hb	MCV	MCH	MCHC	RCDW	Log SF	Log iron	Log TS
Men								
Hb	1.000	−0.009	0.044	0.140	−0.111	0.127	0.156	0.088
MCV		1.000	0.930	−0.074	− 0.001	0.081	0.196	0.210
MCH			1.000	0.297	−0.110	0.118	0.216	0.223
MCHC				1.000	−0.298	0.112	0.077	0.061
RCDW					1.000	−0.123	−0.117	−0.135
Women								
Hb	1.000	0.065	0.101	0.117	−0.072	0.134	0.152	0.110
MCV		1.000	0.934	0.007	−0.143	0.101	0.272	0.291
MCH			1.000	0.360	−0.249	0.088	0.291	0.314
MCHC				1.000	−0.332	−0.020	0.107	0.121
RCDW					1.000	0.028	−0.178	−0.164

Hb was positively correlated with SF, iron and TS in both men and women (0.09 to 0.16). MCV and MCH showed stronger correlations with iron and TS (0.20 to 0.31) than with SF (0.08 to 0.10) in men and women. MCHC showed weak correlations with SF, iron and TS (− 0.02 to 0.12) in men and women. RCDW showed negative correlations with SF, iron and TS (− 0.12 to − 0.14) in men and negative correlations with iron and TS (− 0.16 to − 0.17) but not with SF (0.03) in women.

Estimated hazard ratios for Hb, MCV, MCH, MCHC, and RCDW both as continuous variables and in approximate tertile groups, in relation to cancer incidence and death after adjustment for cancer risk were determined. There were only two instances of possible associations between a haematological parameter and cancer incidence in men or women. There was marginal evidence of an association between MCV (as a continuous variable) and non-skin cancer incidence in women (hazard ratio 1.15, 95% CI 1.013, 1.302; *p* = 0.030) but the hazard ratio was attenuated to non-significance after adjustment for SF, iron and TS (hazard ratio 1.11, 95% CI 0.972, 1.264; *p* = 0.126). There was strong evidence of an association between MCHC and prostate cancer incidence in men; the estimated hazard ratio for an increase of one SD (0.5) in MCHC was 1.27 (95% CI 1.064, 1.507; *p* = 0.008). Men with a MCHC ≤34.1 g/100 ml had a significantly lower risk of prostate cancer compared with men who had a MCHC above this value. Further, these associations remain after further adjustment for SF, iron and TS with the estimated hazard ratio for an increase of one SD (0.5) in MCHC being 1.25 (*p* = 0.014, 95% confidence interval 1.05 to 1.48).

Haematological parameters did not exhibit any association with incidence or death for breast cancer in women or colorectal cancer in women or men. Blood donation was not associated with cancer incidence and death.

## Discussion

To the best of our knowledge, this is the first large-scale population study to investigate the prospective association between haematological parameters as surrogate markers of iron bioavailability, and cancer incidence and death.

In this prospective, observational cohort study, we observed a strong association between MCHC and prostate cancer incidence that remained significant following adjustments for iron markers. There was also a marginal association between MCV and non-skin cancer incidence in women which was attenuated to non-significance after adjustment for iron markers. Hb, MCH and RCDW did not generate any significant association with cancer incidence or death.

We postulate that the overall weak correlation between haematological parameters and serum iron markers in our study may be attributed to the fact that changes in iron levels are reflected to some degree through red blood cell morphology [36, 49, 50]. Factors that could confound the association include diurnal variation in serum iron levels [51–56] and unmeasured changes in iron and haematological parameters in the timeframe between entry into the study and endpoint determination. Several studies have reported positive correlations between serum iron or SF levels with TS, MCV and MCH [57, 58], whilst others have shown no significant correlation between MCV with TS and SF [59, 60].

This study demonstrates consistent trends in associations between increasing iron or haematological parameters and incidence for some cancers. Interestingly, the association between MCHC and prostate cancer was independent of adjustment for iron parameters whilst that of MCV and non-skin cancer in women was dependent on iron. We hypothesise that the variation in our findings can be attributed to the fact that even though iron may contribute to cancer via induction of oxidative stress, changes in the red blood cell parameters may also reflect changes to the body as a result of long term oxidative stress that parallels the drive for carcinogenesis [61–71]. The association between MCHC and prostate cancer which remained significant after iron studies adjustments leads us to speculate that prostate carcinogenesis may be driven by alternative oxidative stress mediators possibly independent of iron. Although many studies have reported an association between oxidative stress and prostate cancer development, inconsistent reports have emerged on the association between iron and prostate carcinogenesis [72–79].

Whilst several studies have reported positive associations between MCHC and oral squamous cell carcinoma [80] or head and neck cancers [81], others found no statistically significant associations [82, 83].

Inconsistent results have been reported regarding the association between MCV and cancer. A retrospective Japanese study found that elevated MCV was associated with the presence of lymphoid and solid organ cancers [84]. In contrast, a study of 253 patients with involuntary weight loss who were investigated for cancer found that MCV was not associated with cancer [85]. We observed an association between MCV, as a continuous variable, and non-skin cancer incidence. A retrospective much larger Korean cohort study of 36,260 cancer-free, non-anaemic men and women found that elevated MCV by quartiles was related to higher all-cause mortality and liver cancer mortality. There was a difference between genders with the highest quartile of MCV (≥95.8 fL) showing higher cancer mortality in men but not in women [86]. A retrospective study by Qu et al. observed that a high MCHC and MCV level was an independent prognostic factor for overall survival in non-small cell lung cancer [87]. We speculate the association between elevation of MCV and cancer in our study may be a consequence of long-term effects of iron-dependent oxidative stress on the red blood cell structure and cancer pathogenesis [86, 88]. This mechanism still remains ambiguous and further research is needed to elucidate this hypothesis.

Substantial variability of findings has been observed in studies that investigated the association between RCDW and various cancers. A positive association have been reported in colon cancer [61, 89] endometrial cancer [90] oesophageal cancer [91–94] renal cell carcinoma [95] or breast cancer [96–98], whilst other studies found no statistically significant associations [85, 99].

There are strengths and limitations of our study. Strengths of our study include a well-defined, community-based cohort with a long follow-up period and adjustment of associations for a wide range of potential confounders. We excluded patients who were using erythropoeisis-stimulating agents that can affect these hematological parameters. Weaknesses include the relatively limited cohort size, which may have limited power, and the limited number of time-points during the course of the follow-up. Furthermore, we tested five haematological parameters for five cancers in men and women raising the possibility of false positive findings. Hence, the significance of the discovered possible associations between MCV and non-skin cancer incidence in women (*p* = 0.030) and MCHC and prostate cancer incidence in men (*p* = 0.008) should be regarded with care. In the current study, data on potential confounders for specific cancer risk factors, such as intake of red meat, fish, fibre, saturated fat and vitamin and mineral levels were unavailable and thus may also contribute to the inconsistent finding with those of previously published studies. As study subjects self-reported their histories of smoking and alcohol use during their participation in the survey, this may also limit accuracy of data. The nature of any pathophysiological process effecting haematological parameters decades before the occurrence of cancer and which contributes to cancer development remains unclear.

## Conclusions

In conclusion, both MCHC and MCV were associated with cancer incidence in a Western Australian population, although only MCHC remained associated with prostate cancer incidence after adjusting with serum iron and TS (circulating iron) and SF (storage iron). Haematological parameters are thus of limited utility in population profiling for future cancer risk.

## Abbreviations

ALT: Alanine transaminase
BMI: Body mass index
C-RP: C-reactive protein
GGT: Gamma-glutamyl transferase
Hb: Haemoglobin
HDL: High density lipoprotein
MCH: Mean cell haemoglobin
MCHC: Mean corpuscular haemoglobin concentration
MCV: Mean corpuscular volume
RCDW: Red cell distribution width
SF: Serum ferritin
TS: Transferrin saturation

## References

[1] Waldvogel-Abramowski S, Waeber G, Gassner C, Buser A, Frey BM, Favrat B (2014). Physiology of Iron metabolism. Transfus Med Hemother.

[2] Abbaspour N, Hurrell R, Kelishadi R (2014). Review on iron and its importance for human health. J Res Med Sci.

[3] Lieu PT, Heiskala M, Peterson PA, Yang Y (2001). The roles of iron in health and disease. Mol Asp Med.

[4] Beard JL, Dawson HD, O’Dell BL, Sunde RA (1997). Iron. Handbook of nutritionally essential mineral elements.

[5] Stauder R, Valent P, Theurl I. Anemia at older age: etiologies, clinical implications and management. Blood. 2017; 10.1182/blood-2017-07-746446. 10.1182/blood-2017-07-746446 29141943

[6] Theurl I, Aigner E, Theurl M, Nairz M, Seifert M, Schroll A, Sonnweber T, Eberwein L, Witcher DR, Murphy AT, Wroblewski VJ, Wurz E, Datz C, Weiss G (2009). Regulation of iron homeostasis in anemia of chronic disease and iron deficiency anemia: diagnostic and therapeutic implications. Blood.

[7] Jankowska EA, Rozentryt P, Witkowska A, Nowak J, Hartmann O, Ponikowska B, Borodulin-Nadzieja L, Banasiak W, Polonski L, Filippatos G, McMurray JJ, Anker SD, Ponikowski P (2010). Iron deficiency: an ominous sign in patients with systolic chronic heart failure. Eur Heart J.

[8] Yerlikaya A, Bulbul MC, Afsar B, Dagel T, Aslan G, Voroneanu L, Siriopol D, Covic A, Kanbay M. Iron in kidney and heart failure: from theory to practice. Int Urol Nephrol. 2017; 10.1007/s11255-017-1708-6. 10.1007/s11255-017-1708-6 28940112

[9] Earley CJ, Allen RP, Beard JL, Connor JR (2000). Insight into the pathophysiology of restless legs syndrome. J Neurosci Res.

[10] Trenkwalder C, Allen R, Hogl B, Paulus W, Winkelmann J (2016). Restless legs syndrome associated with major diseases: a systematic review and new concept. Neurology.

[11] Pietrangelo A (2004). Hereditary hemochromatosis—a new look at an old disease. N Engl J Med.

[12] Grosse SD, Gurrin LC, Bertalli NA, Allen KJ. Clinical penetrance in hereditary hemochromatosis: estimates of the cumulative incidence of severe liver disease among HFE C282Y homozygotes. Genet Med. 2017; 10.1038/gim.2017.121. 10.1038/gim.2017.121 PMC5797490 28771247

[13] Sikorska K, Bernat A, Wroblewska A (2016). Molecular pathogenesis and clinical consequences of iron overload in liver cirrhosis. Hepatobiliary Pancreat Dis Int.

[14] Kawabata H. The mechanisms of systemic iron homeostasis and etiology, diagnosis, and treatment of hereditary hemochromatosis. Int J Hematol. 2017; 10.1007/s12185-017-2365-3. 10.1007/s12185-017-2365-3 29134618

[15] Murphy CJ, Oudit GY (2010). Iron-overload cardiomyopathy: pathophysiology, diagnosis, and treatment. J Card Fail.

[16] Tauchenová L, Křížová B, Kubánek M, Fraňková S, Melenovský V, Tintěra J, et al. Successful treatment of iron-overload cardiomyopathy in hereditary hemochromatosis with deferoxamine and deferiprone. Can J Cardiol. 2016; 10.1016/j.cjca.2016.07.589. 10.1016/j.cjca.2016.07.589 27789107

[17] Melchiori L, Gardenghi S, Beta-Thalassemia RS. HiJAKing ineffective erythropoiesis and Iron overload. Adv Hematol. 2010; 10.1155/2010/938640. 10.1155/2010/938640 PMC2873658 20508726

[18] Mishra AK, Tiwari A (2013). Iron overload in Beta Thalassaemia major and intermedia patients. Maedica.

[19] Porter J, Garbowski M. Consequences and management of iron overload in sickle cell disease. Hematology Am Soc Hematol Educ Program. 2013;2013:447–56. 10.1182/asheducation-2013.1.447 24319218

[20] Darbari DS, Kple-Faget P, Kwagyan J, Rana S, Gordeuk VR, Castro O. Circumstances of death in adult sickle cell disease patients. Am J Hematol. 2006;81(11):858–63. 10.1002/ajh.20685 16924640

[21] Toyokuni S (2009). Role of iron in carcinogenesis: cancer as a ferrotoxic disease. Cancer Sci.

[22] Lenarduzzi M, Hui AB, Yue S, Ito E, Shi W, Williams J, et al. Hemochromatosis enhances tumor progression via upregulation of intracellular iron in head and neck cancer. PLoS One. 2013; 10.1371/journal.pone.0074075. 10.1371/journal.pone.0074075 PMC3753261 23991213

[23] Lagergren K, Wahlin K, Mattsson F, Alderson D, Lagergren J (2016). Haemochromatosis and gastrointestinal cancer. Int J Cancer.

[24] Shaheen NJ, Silverman LM, Keku T, Lawrence LB, Rohlfs EM, Martin CF (2003). Association between hemochromatosis (HFE) gene mutation carrier status and the risk of colon cancer. J Natl Cancer Inst.

[25] Chua AC, Knuiman MW, Trinder D, Divitini ML, Olynyk JK (2016). Higher concentrations of serum iron and transferrin saturation but not serum ferritin are associated with cancer outcomes. Am J Clin Nutr.

[26] Wu T, Sempos CT, Freudenheim JL, Muti P, Smit E (2004). Serum iron, copper and zinc concentrations and risk of cancer mortality in US adults. Ann Epidemiol.

[27] Wen CP, Lee JH, Tai YP, Wen C, Wu SB, Tsai MK (2014). High serum iron is associated with increased cancer risk. Cancer Res.

[28] Zacharski LR, Chow BK, Howes PS, Shamayeva G, Baron JA, Dalman RL (2008). Decreased cancer risk after iron reduction in patients with peripheral arterial disease: results from a randomized trial. J Natl Cancer Inst.

[29] Kato J, Miyanishi K, Kobune M, Nakamura T, Takada K, Takimoto R (2007). Long-term phlebotomy with low iron diet therapy lowers risk of development of hepatocellular carcinoma from hepatitis C. J Gastroenterol.

[30] Chaston TB, Lovejoy DB, Watts RN, Richardson DR (2003). Examination of the antiproliferative activity of iron chelators: multiple cellular targets and the different mechanism of action of triapine compared with desferrioxamine and the potent pyridoxal isonicotinoyl hydrazone analogue 311. Clin Cancer Res.

[31] Darnell G, Richardson DR (1999). The potential of iron chelators of the pyridoxal isonicotinoyl hydrazone class as effective antiproliferative agents III: the effect of the ligands on molecular targets involved in proliferation. Blood.

[32] Le NT, Richardson DR (2004). Iron chelators with high antiproliferative activity up-regulate the expression of a growth inhibitory and metastasis suppressor gene: a link between iron metabolism and proliferation. Blood.

[33] Hann HW, Stahlhut MW, Blumberg BS (1988). Iron nutrition and tumor growth: decreased tumor growth in iron-deficient mice. Cancer Res.

[34] Jiang Y, Xue ZH, Shen WZ, Du KM, Yan H, Yu Y (2005). Desferrioxamine induces leukemic cell differentiation potentially by hypoxia-inducible factor-1 alpha that augments transcriptional activity of CCAAT/enhancer-binding protein alpha. Leukemia.

[35] Mascitelli L, Pezzetta F, Sullivan JL (2010). Aspirin-associated iron loss as an anticancer mechanism. Med Hypotheses.

[36] Kautz L, Nemeth E (2014). Molecular liaisons between erythropoiesis and iron metabolism. Blood.

[37] Yehuda S, Mostofsky DI. Iron Deficiency and Overload: from basic biology to clinical medicine. 2010. New York: Humana Press. P165–167, 307.

[38] Lee GR. Iron deficiency and iron-deficiency anemia. In: Lee GR, Foerster J, Lukons J, Paraskevas F, Greer JP, Rodgers GM, editors. Wintrobe's clinical Haematology. Baltimore 10^th^ ed: Williams & Wilkins; 1999. p. 979–1010.

[39] Barton JC, Bertoli LF, Rothenberg BE (2000). Peripheral blood erythrocyte parameters in hemochromatosis: evidence for increased erythrocyte hemoglobin content. J Lab Clin Med.

[40] Rossi E, Olynyk JK, Cullen DJ, Papadopoulos G, Bulsara M, Summerville L, Powell LW (2000). Compound heterozygous hemochromatosis genotype predicts increased iron and erythrocyte indices in women. Clin Chem.

[41] Datz C, Haas T, Rinner H, Sandhofer F, Patsch W, Paulweber B (1998). Heterozygosity for the C282Y mutation in the hemochromatosis gene is associated with increased serum iron, transferrin saturation, and hemoglobin in young women: a protective role against iron deficiency?. Clin Chem.

[42] Medicare Item Reports. Medicareaustralia.gov.au. 2017. http://medicarestatistics.humanservices.gov.au/statistics/mbs_item.jsp. Accessed 19 Nov 2017.

[43] Medicare Benefits Schedule Book Category 6. Australian Government Department of Health. 2013. Available from online ISBN: 978-1-74186-061-0.

[44] M. Greeff. Laboratory bulletin. Alberta Health Services. 2016:2016. https://www.albertahealthservices.ca/. Accessed 19 Nov 2017.

[45] Laboratory testing guidelines. Waikato District Health Board. August 2015:2015. https://lab.waikatodhb.health.nz/. Accessed 19 Nov 2017.

[46] Fox CJ, Cullen DJ, Knuiman MW, Cumpston GN, Divitini ML. Rossi et al. Effects of body iron stores and haemochromatosis genotypes on coronary heart disease outcomes in the Busselton health study. J Cardiovasc Risk. 2002;9(5):287–93. 10.1177/174182670200900510 12394323

[47] Hung J, Knuiman MW, Divitini ML, Davis T, Beilby JP (2008). Prevalence and risk factor correlates of elevated C-reactive protein in an adult Australian population. Am J Cardiol.

[48] D'Arcy C, Holman CD, Bass AJ, Rouse IL, Hobbs MS. Population-based linkage of health records in Western Australia: development of a health services research linked database. Aust N Z J Public Health. 1999;23(5):453–9. 10.1111/j.1467-842x.1999.tb01297.x 10575763

[49] von Drygalski A, Adamson JW (2013). Iron metabolism in man. JPEN J Parenter Enteral Nutr.

[50] Hassan R, Abdullah WZ, Nik Hussain NH (2005). Anemia and iron status of Malay women attending an antenatal clinic in Kubang Kerian, Kelantan. Malaysia Am J Obstet Gynecol.

[51] Hoyer K (1944). Physiologic variations in the iron content of human blood serum. Acta Med Scand.

[52] Hamilton LD, Gubler CJ, Cartwright GE, Wintrobe MM (1950). Diurnal variation in plasma iron level of man. Proc Soc Exp Biol Med.

[53] Statland BE, Winkel P, Bokelund H (1976). Variation of serum iron concentration in young healthy men: within-day and day-today changes. Clin Biochem.

[54] Wiltink WF, Kruithof J, Mol C, Bos MG, van Eijk HG (1973). Diurnal and nocturnal variations of the serum iron in normal subjects. Clin ChimActa.

[55] Bowie EJ, Tauxe WN, Sjobergew WE, Yamaguchi MY (1963). Daily variation in the concentration of iron in serum. Am J Clin Pathol.

[56] Dale JC, Burritt MF, Zinsmeister AR (2002). Diurnal variation of serum iron, iron-binding capacity, transferrin saturation, and ferritin levels. Am J Clin Pathol.

[57] Tomkiewicz-Pajak L, Plazak W, Kolcz J, Pajak J, Kopec G, Dluzniewska N (2014). Iron deficiency and hematological changes in adult patients after Fontan operation. J Cardiol.

[58] McLaren CE, Barton JC, Gordeuk VR, Wu L, Adams PC, Reboussin DM (2007). Determinants and characteristics of mean corpuscular volume and hemoglobin concentration in white HFE C282Y homozygotes in the hemochromatosis and iron overload screening study. Am J Hematol.

[59] Hastka J, Lasserre JJ, Schwarzbeck A, Reiter A, Hehlmann R (1996). Laboratory tests of iron status: correlation or common sense?. Clin Chem.

[60] Mikhail S, Phatak P (2009). Elevated mean corpuscular volume in patients with hereditary hemochromatosis. Comp Clin Pathol.

[61] Ay S, Eryilmaz MA, Aksoy N, Okus A, Unlu Y, Sevinc B (2015). Is early detection of colon cancer possible with red blood cell distribution width?. Asian Pac J Cancer Prev.

[62] Petersen DR (2005). Alcohol, iron-associated oxidative stress, and cancer. Alcohol.

[63] Darash-Yahana M, Pozniak Y, Lu M, Sohn YS, Karmi O, Tamir S (2016). Breast cancer tumorigenicity is dependent on high expression levels of NAF-1 and the lability of its Fe-S clusters. Proc Natl Acad Sci U S A.

[64] Bae YJ, Yeon JY, Sung CJ, Kim HS, Sung MK (2009). Dietary intake and serum levels of iron in relation to oxidative stress in breast cancer patients. J Clin Biochem Nutr.

[65] Hattangadi SM, Lodish HF (2007). Regulation of erythrocyte lifespan: do reactive oxygen species set the clock?. J Clin Investig.

[66] O Ayanshina, S Adeola, M Igwo-Ezikpe. Levels of some Oxidative Stress Biomarkers and Hematological Parameters in Cancer Patients from Lagos, Nigeria. FASEB J 2015;29(1) Suppl 576.4 doi: 10.1096/fj.1530-6860.

[67] Patel KV, Semba RD, Ferrucci L, Newman AB, Fried LP, Wallace RB (2010). Red cell distribution width and mortality in older adults: a meta-analysis. J Gerontol A Biol Sci Med Sci.

[68] Agarval S (2012). Red cell distribution width, inflammatory markers and cardiorespiratory fitness: results from the National Health and nutrition examination survey. Indian Heart J.

[69] Ghaffari S (2008). Oxidative stress in the regulation of normal and neoplastic hematopoiesis. Antioxid Redox Signal.

[70] Zhao B, Mei Y, Yang J, Ji P (2016). Erythropoietin-regulated oxidative stress negatively affects enucleation during terminal erythropoiesis. Exp Hematol.

[71] Waggiallah H, Alzohairy M (2011). The effect of oxidative stress on human red cells glutathione peroxidase, glutathione reductase level, and prevalence of anemia among diabetics. N Am J Med Sci.

[72] Khandrika L, Kumar B, Koul S, Maroni P, Koul HK (2009). Role of oxidative stress in prostate Cancer. Cancer Lett.

[73] Kumar B, Koul S, Khandrika L, Meacham RB, Koul HK (2008). Oxidative stress is inherent in prostate cancer cells and is required for aggressive phenotype. Cancer Res.

[74] Sikka SC (2003). Role of oxidative stress response elements and antioxidants in prostate cancer pathobiology and chemoprevention--a mechanistic approach. Curr Med Chem.

[75] Oh B, Figtree G, Costa D, Eade T, Hruby G, Lim S, Elfiky A, Martine N, Rosenthal D, Clarke S, Back M (2016). Oxidative stress in prostate cancer patients: a systematic review of case control studies. Prostate International.

[76] Knekt P, Reunanen A, Takkunen H, Aromaa A, Heliövaara M, Hakulinen T (1994). Body iron stores and risk of cancer. Int J Cancer.

[77] Stevens RG, Graubard BI, Micozzi MS, Neriishi K, Blumberg BS (1994). Moderate elevation of body iron level and increased risk of cancer occurrence and death. Int J Cancer.

[78] Kuvibidila SR, Gauthier T, Rayford W (2004). Serum ferritin levels and transferrin saturation in men with prostate cancer. J Natl Med Assoc.

[79] Wang X, An P, Zeng J, Liu X, Wang B, Fang X (2017). Serum ferritin in combination with prostate-specific antigen improves predictive accuracy for prostate cancer. Oncotarget.

[80] Bhattathiri VN (2001). Relation of erythrocyte and iron indices to oral cancer growth. Radiother Oncol.

[81] Bhattacharjee A, Borah FR, Sarbani G, Devnath B, S U (2015). Evaluation of hematological parameters as a possible marker for head-and-neck cancer and precancerous conditions. J Evol Med Dent Sci.

[82] Anees Ahmed RA, Ganvir SM, Hazarey VK (2014). Relation of erythrocyte indices and serum iron level with clinical and histological progression of oral squamous cell carcinoma in Central India. J Investig Clin Dent.

[83] Miller A, Chodos RB, Emerson CP, Ross JF (1956). Studies of the anaemia and iron metabolism in cancer. J Clin Investig.

[84] Takahashi N, Kameoka J, Takahashi N, Tamai Y, Murai K, Honma R (2016). Causes of macrocytic anemia among 628 patients: mean corpuscular volumes of 114and 130 f. As critical markers for categorization. Int J Hematol.

[85] Baicus C, Caraiola S, Rimbas M, Patrascu R, Baicus A (2011). Utility of routine hematological and inflammation parameters for the diagnosis of cancer in involuntary weight loss. J Investig Med.

[86] Yoon HJ, Kim K, Nam YS, Yun JM, Park M (2016). Mean corpuscular volume levels and all-cause and liver cancer mortality. Clin Chem Lab Med.

[87] Qu X, Zhang T, Ma H, Sui P, Du J (2014). Lower mean corpuscular hemoglobin concentration is associated with unfavorable prognosis of resected lung cancer. Future Oncol.

[88] Tsantes AE, Bonovas S, Travlou A, Sitaras NM (2006). Redox imbalance, macrocytosis, and RBC homeostasis. Antioxid Redox Signal.

[89] Spell DW, Jones DV, WF David H, Bessman J (2004). The value of a complete blood count in predicting cancer of the colon. Cancer detect Prev. Cancer Detect Prev.

[90] Kemal Y, Demirag G, Baş B, Önem S, Teker F, Yücel İ (2015). The value of red blood cell distribution width in endometrial cancer. Clin Chem Lab Med.

[91] Sun P, Zhang F, Chen C, Bi X, Yang H, An X (2016). The ratio of hemoglobin to red cell distribution width as a novel prognostic parameter in esophageal squamous cell carcinoma: a retrospective study from southern China. Oncotarget.

[92] Wan GX, Chen P, Cai XJ, Li LJ, Yu XJ, Pan DF (2016). Elevated red cell distribution width contributes to a poor prognosis in patients with esophageal carcinoma. Clin Chim Acta.

[93] Hirahara N, Matsubara T, Kawahara D, Mizota Y, Ishibashi S, Tajima Y (2016). Prognostic value of hematological parameters in patients undergoing esophagectomy for esophageal squamous cell carcinoma. Int J Clin Oncol.

[94] Chen G-P, Huang Y, Yang X, Feng J-F. A nomogram to predict prognostic value of red cell distribution width in patients with esophageal Cancer. Mediat Inflamm. 2015; 10.1155/2015/854670. 10.1155/2015/854670 PMC4633693 26578822

[95] Wang FM, Xu G, Zhang Y, Ma LL. Red cell distribution width is associated with presence, stage, and grade in patients with renal cell carcinoma. Dis Markers. 2014; 10.1155/2014/860419. 10.1155/2014/860419 PMC4280806 25580051

[96] Huang DP, Ma RM, Xiang YQ. Utility of red cell distribution width as a prognostic factor in young breast Cancer patients. Medicine (Baltimore). 2016; 10.1097/MD.0000000000003430. 10.1097/MD.0000000000003430 PMC4998693 27124030

[97] Wang C, Civan J, Lai Y, Cristofanilli M, Hyslop T, Palazzo JP (2015). Racial disparity in breast cancer survival: the impact of pre-treatment hematologic variables. Cancer Causes Control.

[98] Yao M, Liu Y, Jin H, Liu X, Lv K, Wei H (2014). Prognostic value of preoperative inflammatory markers in Chinese patients with breast cancer. Onco Targets Ther.

[99] Speights VO, Johnson MW, Stoltenberg PH, Rappaport ES, Helbert B, Riggs MW. Complete blood count indices in colorectal carcinoma. Arch Pathol Lab Med. 1992;116(3):258–60. 1536610

